# The surgical wound in infrared: thermographic profiles and early stage test-accuracy to predict surgical site infection in obese women during the first 30 days after caesarean section

**DOI:** 10.1186/s13756-018-0461-7

**Published:** 2019-01-07

**Authors:** Charmaine Childs, Nicola Wright, Jon Willmott, Matthew Davies, Karen Kilner, Karen Ousey, Hora Soltani, Priya Madhuvrata, John Stephenson

**Affiliations:** 1 Faculty of Health and Wellbeing, Montgomery House, Sheffield Hallam University, 32 Collegiate Crescent, Sheffield, S102BP England; 2 Department of Electronic and Electrical Engineering, Portobello Centre, University of Sheffield, Sheffield, S1 4ET England; 3 Sheffield Hallam University, 32 Collegiate Crescent, Sheffield, S10 2BP England; 4 Institute of Skin Integrity and Infection Prevention, University of Huddersfield, Queensgate, Huddersfield, HD1 3DH England; 5 Faculty of Health and Wellbeing, Sheffield Hallam University, 32 Collegiate Crescent, Sheffield, S10 2BP England; 6 Obstetrics and Gynaecology, Jessop Hospital, Tree Root Walk, Sheffield, S10 2SF England; 7 Biomedical Statistics, University of Huddersfield, Queensgate, Huddersfield, HD1 3DH England

**Keywords:** Surgical site infection, Obesity, Caesarean section, Prognosis, Antibiotics, Infrared thermography, Thermal mapping

## Abstract

**Background:**

Prophylactic antibiotics are commonly prescribed intra-operatively after caesarean section birth, often at high doses. Even so, wound infections are not uncommon and obesity increases the risk. Currently, no independent wound assessment technology is available to stratify women to low or high risk of surgical site infection (SSI).

Study Aim: to investigate the potential of non-invasive infrared thermography (IRT), performed at short times after surgery, to predict later SSI.

**Methods:**

IRT was undertaken in hospital on day 2 with community follow up (days 7, 15, 30) after surgery. Thermal maps of wound site and abdomen were accompanied by digital photographs, the latter used for wound assessment by six experienced healthcare professionals. Confirmatory diagnosis of SSI was made on the basis of antibiotic prescribing by the woman’s community physician with logistic regression models derived to model dichotomous outcomes.

**Results:**

Fifty-three women aged 21–44 years with BMI 30.1–43.9 Kg.m^− 2^ were recruited. SSI rate (within 30 days) was 28%. Inter-rater variability for ‘professional’ opinion of wound appearance showed poor levels of agreement. Two regions of interest were interrogated; wound site and abdomen. Wound site temperature was consistently elevated (1.5 °C) above abdominal temperature with similar values at days 2,7,15 in those who did and did not, develop SSI. Mean abdominal temperature was lower in women who subsequently developed SSI; significantly so at day 7. A unit (1 °C) reduction in abdominal temperature was associated with a 3-fold raised odds of infection. The difference between the sites (wound minus abdomen temperature) was significantly associated with odds of infection; with a 1 °C widening in temperature associated with an odds ratio for SSI of 2.25 (day 2) and 2.5 (day 7). Correct predictions for wound outcome using logistic regression models ranged from 70 to 79%;

**Conclusions:**

IRT imaging of wound and abdomen in obese women undergoing c-section improves upon visual (subjective) wound assessment. The proportion of cases correctly classified using the wound-abdominal temperature differences holds promise for precision and performance of IRT as an independent SSI prognostic tool and future technology to aid decision making in antibiotic prescribing.

## Introduction

In the global context of hospital-acquired infections (HAIs), recent work by the World Health Organisation (WHO) shows that in low- and middle-income countries, surgical site infection (SSI) is the most widely surveyed and frequent HAI, affecting 33% of patients undergoing surgical procedures [1]. In Europe and the United States of America SSI is the second most frequent HAI [2], and remains a substantial cause of post-operative morbidity and financial burden for health systems [3]. Yet SSI is preventable, given adequate means for surveillance, prevention and early diagnosis.

SSI attracts attention from national [4] and international organisations [1] with publications for guidelines on prevention and consensus for best practice [5] but there are groups within our international community who are increasingly recognised as being at particular risk. Across most surgical specialities (e.g. [6–8] obesity emerges consistently as a significant risk factor for SSI.

The growing ‘epidemic’ of obesity, particularly in women of reproductive age presents a major problem for maternal health [9]. Over the last 30 years, the proportion of births delivered by caesarean section (c-section) has risen. Globally, c-section rates are high in Latin-America at 40.5% with America and Oceania at 32.3 and 31.1% respectively [10] and with older women having the highest rates [11]. In the UK in 2017 c-section rate was 25% [12]. Obesity (body mass index, BMI > 30 kg.m^− 2^) increases the need for c-section birth and obese women giving birth by c-section are at a higher risk of SSI [13].

Whilst SSI rates have been reported as 5.5 to 7.5% for elective and emergency c-section respectively [14], even higher rates are also reported at 9.6% [15]. Others report rates of 4–9% depending on the surveillance methods used for identifying infection [16, 17]. More recently, in a retrospective series of 400 women in South East Asia, prevalence of SSI in a cohort with mixed body mass index (BMI) reached 18.8% [18]. The greatest risk for SSI after c-section is obesity [19] and more so for morbidly obese (BMI ≥40 kg.m^− 2^) women, where the SSI rate can reach 50% [20].

With the trend towards short hospital stays after childbirth [21] (including birth by c-section) the management of post-operative wound infections, when they occur, are increasingly a healthcare problem which develops in the community. Typically c-section infections are superficial [22], but occasionally, bacteria infiltrate deeper tissue and organ spaces. Catastrophic clinical deterioration leading to severe tissue necrosis sepsis and death [23] is not a common occurrence after childbirth, but in the light of its impact on the quality of life and adverse experiences of mothers and their families, severe infection after c-section is becoming a growing concern among health care providers and policy makers [24].

In view of the burgeoning problems in society of obesity, the increasing numbers of babies delivered by c-section and the link between c-section birth and SSI, there is a real need to improve wound surveillance in the interim between hospital and community, for it is in the community, rather than in the hospital, that problems with wound healing, wound breakdown and wound infections become apparent. Currently there is no *wound imaging diagnostic* available in clinical practice with which to forewarn of early SSI risk or later wound breakdown.

In previous studies [25, 26] we have observed a wound ‘signature’ on infrared thermography which holds promise as an early diagnostic biomarker to forewarn of later delayed healing and SSI. In the present study we have tested the concept of thermographic mapping of the surgical wound and temporal thermal profiles of the abdomen and wound site in the visible, *and* in the infrared (IR) spectrum; the primary aim being to establish the characteristics of the abdomen and wound site in infrared to aid stratification of obese women to risk for later SSI. The study objectives were threefold: to 1) explore concordance in visual wound assessment between observers; 2) document the temporal infrared profile of the abdomen *and* c-section wound during the course of healing; and 3) show early-stage performance and test-accuracy in a pilot study of the performance of infrared thermography signatures to predict later SSI.

## Methods

### Study design

This study was undertaken as a prospective observational thermal mapping and *early-stage* test-accuracy investigation. It involved comparison of visual wound assessments by clinicians, and provided temporal mapping of wound healing after c-section with provisional information on thermography-based SSI risk stratification for obese women postpartum.

In this study, thermal ‘mapping’ was conducted during the SSI surveillance period defined by the Centre for Disease Control (CDC) as wound infection occurring within 30 days after surgery [27].

### Ethics approval

The study received all required institutional research ethics and governance approvals: research ethics committee, health research authority, NHS Trust research governance and University research ethics. All approvals were in place at the time of the commencement of the study. The study also received approvals and clinical support via the regional Clinical Research Network (CRN). All identifiable and non-identifiable data, including thermal images were stored and retained in accordance with the data protection Act (1998). Informed consent was obtained from all participants.

### Participants

Women with a booking BMI ≥30 kg⋅m^− 2^ who had delivered an infant by elective or emergency c-section were eligible to participate in the study. Women with a negative pressure wound therapy (NPWT) dressing in situ on return to the post-natal ward were excluded as IR thermal imaging cannot be undertaken with occlusive dressings in situ.

Screening for eligibility was undertaken by a research midwife or nurse of the National Institute for Health Research (NIHR) CRN in the antenatal clinics. Women were provided with a participant information sheet and given the time to consider the invitation to participate in the study. Confirmation of participation was undertaken on the postnatal wards after the birth of the baby. Once the baby had been delivered, women were approached once more with full information, written and verbal, to ensure that they were comfortable to continue to participate in the study.

Participation involved one thermal imaging session during the hospital stay (typically 24–48 h after the birth) and three further imaging follow-up sessions at the woman’s own home targeted to days 7, 15 and 30 postpartum.

### Sample size

Recruiting to time available for this study i.e. over 10 months, and an estimated SSI incidence of 20%, the thermal imaging signature for early stage SSI test-accuracy would be expected to correctly identify eight of 10 women with a SSI (sensitivity) and correctly identify 32 out of 40 who do not develop an infection (specificity) with a study sample of 50 women.

### Demographics

Study information gathered included name, age, pregnancy history (gravida, parity), early pregnancy weight and height, operative procedure and wound closure methods. To stratify BMI, obese (type 1: 30 kg⋅m^− 2^ ≤ BMI < 35 kg⋅m^− 2^); severe obesity (type 2: 35 kg⋅m^− 2^ ≤ BMI < 40 kg⋅m^− 2^) and morbid obesity (type 3: BMI ≥40 kg⋅m^− 2^) categories were used. Clinical information was obtained for body temperature from the last recorded clinical measurement before imaging. Information of medications (including antibiotic regimen), blood loss (ml), smoking status and pre-existing co-morbidity was obtained from the maternal records.

### Antibiotic prophylaxis and wound screening

Local antibiotic prophylaxis follows a protocol for intraoperative (before skin incision) intravenous antibiotic administration with cefuroxime 1.5G, metronidazole 500 mg. Post-operative (oral) antibiotic prophylaxis, predominately co-amoxyclav (500/125 amoxicillin/ clavulanic acid) is given for 5 days in women with an early BMI ≥ 40 kg⋅m^− 2^. Postoperative oral antibiotics are also prescribed in the event of a clinical indication or concern for infection. Wound swabs were taken at the discretion of the clinical team (in-patients) or by the general practitioner (GP) in the event that the women returned to the GP with suspicion of wound infection.

### Ambient conditions

Before undertaking thermal imaging, measurements of ambient conditions; air temperature (°C) relative humidity (RH%) air velocity (m⋅s^− 1^) were taken with a hand-held weather meter (Kestrel 3000, Richard Paul Russell Ltd., Hampshire UK). Measurements were made at the hospital bedside and at each home visit.

### Wellness screen and wound infection (at home follow-up)

At each home visit women were asked a series of questions to establish overall health and the personal views of the healing progress of their c-section wound. Broadly, this included the woman’s general health since the birth of the baby and whether there had been episodes of illness (including fever). With regard to the scar and clinical evidence for wound infection, the CDC criterion was used, and responses documented as an SSI assessment on day 7. The CDC criteria were used again as a guide for wound progress on days 15 and 30. Information sought included episodes of pain in or around the wound site, haematoma, signs of inflammation (redness, swelling, heat) and/or exudate (purulent or serous fluid) malodour, discolouration (in or around the wound) and including evidence for early indications of wound breakdown. In addition, notes were made at each visit of the appearance of scar and surrounding tissue. Women were also asked about wound cleansing methods. In the event of antibiotic therapy, the type and dose prescribed was noted.

Follow-up was undertaken by sending a Fax to the GP clinic with a request to return a short questionnaire regarding clinic attendance, antibiotics prescribed (type, dose, duration) and whether a clinical diagnosis of wound infection had been made within the first 30 days after c-section. Wound swab reports were obtained via hospital record systems.

### Thermal imaging of skin surface

For c-section surgery, a lower abdominal, surgical transverse incision was performed in all women using the method described by Pfannenstiel [28]. This is preferred for its cosmetic advantage, with the curve of the incision in a natural fold of skin. After surgical closure of the uterus and rectus sheath, skin closure was achieved using absorbable subcutaneous sutures. In some obese women the large abdominal “overhang” or pannus obscured the wound site. To image the wound, the abdomen was lifted upwards to ‘expose’ the surgical site.

### Imaging protocol

In hospital, thermal imaging was undertaken after removal of dry dressing and with women lying supine. As we described previously [25], all extraneous sources of IR radiation were minimised. Images were taken 15 min after the camera was switched on, and after the measurement and recording of ambient conditions had been recorded.

In the hospital setting, any dry dressing covering the wound was removed. In the home, clothing was folded away from the field of view (FOV) and the pubic region below the scar line covered with a sheet. During follow-up visits at home, women selected either their own bed or a sofa. If a supine position could be achieved, and in view of recent surgery, women were requested to lie as flat as comfortable.

**Fig. 1:**
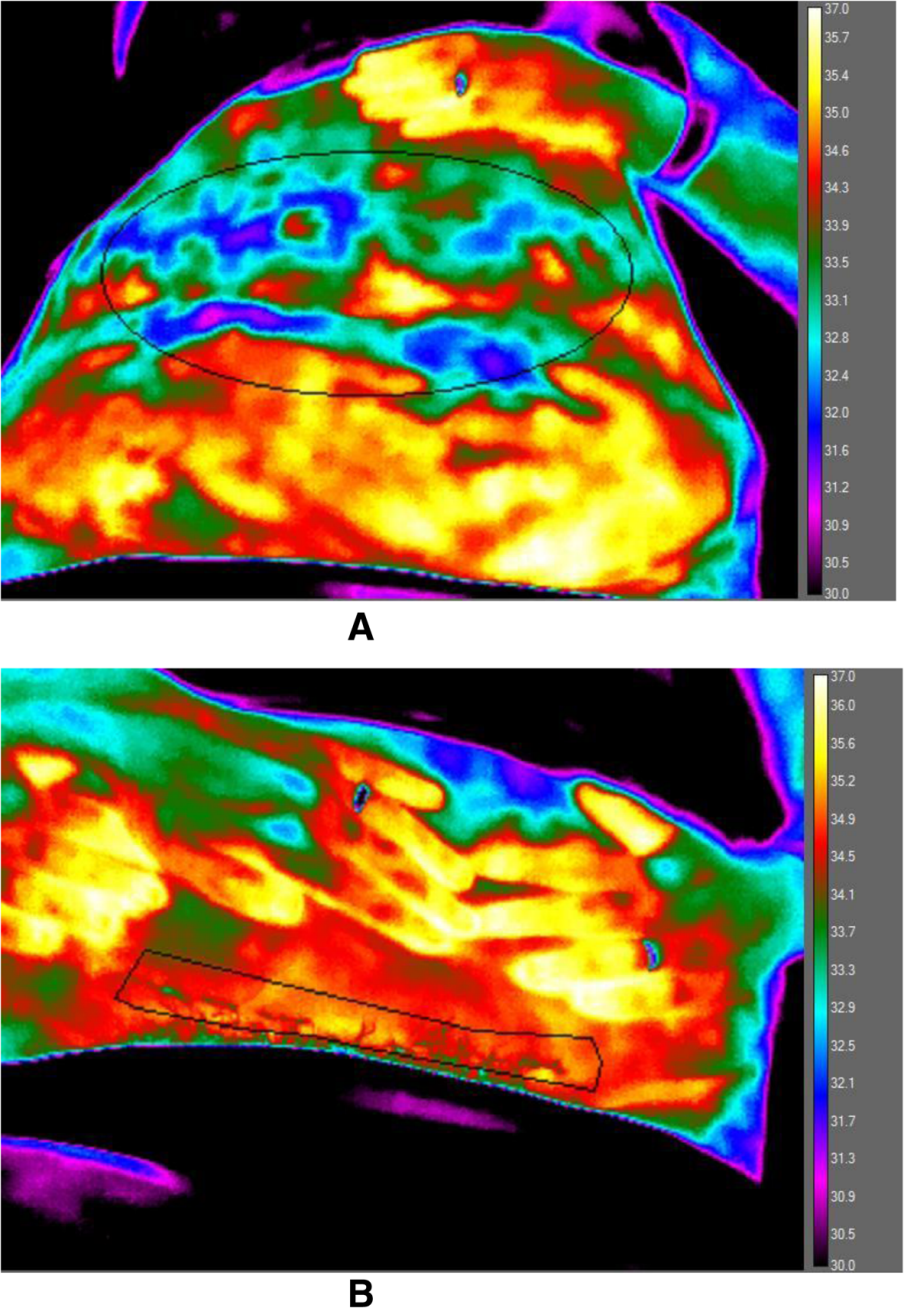
Abdominal thermal maps showing regions of interest (ROIs) with scale of abdominal temperature set to 30-37 °C. Upper panel (a) ROI 1 is of the abdomen with umbilicus centrally; ROI 2 (lower panel,b) shows the region of the scar and surrounding site

Two abdominal regions of interest (ROI) were selected. The first ROI focused on the central abdominal region (umbilicus centrally, ROI 1, Fig. 1a) and before lifting the pannus. The second ROI (ROI 2,) included the full length of the c-section wound and immediate surrounding skin after lifting the pannus (Fig. 1b).

For consistency of the images, the c-section wound ROI 2 (Fig. 1b) was bounded by right and left iliac regions visible in the field of view. Wherever possible, the c-section wound was exposed to air for 10 min before imaging. Where hair was visible, these structures served to aid image focus. Images were taken from: a) the foot of the bed or sofa; and b) from an angle of approximately 45° over the abdomen to give a downward focus. Three to six consecutive images were taken for each ROI.

### Equipment and calibration

Thermal imaging was undertaken using a portable thermal camera (T450sc, uncooled microbolometer, FLIR Täby, Sweden) with image resolution of 320 × 240 pixels. A separate digital photograph was taken focusing directly on the abdomen and scar and to assist in visual inspection of the wound site.

### Measurement reliability

**Fig. 2:**
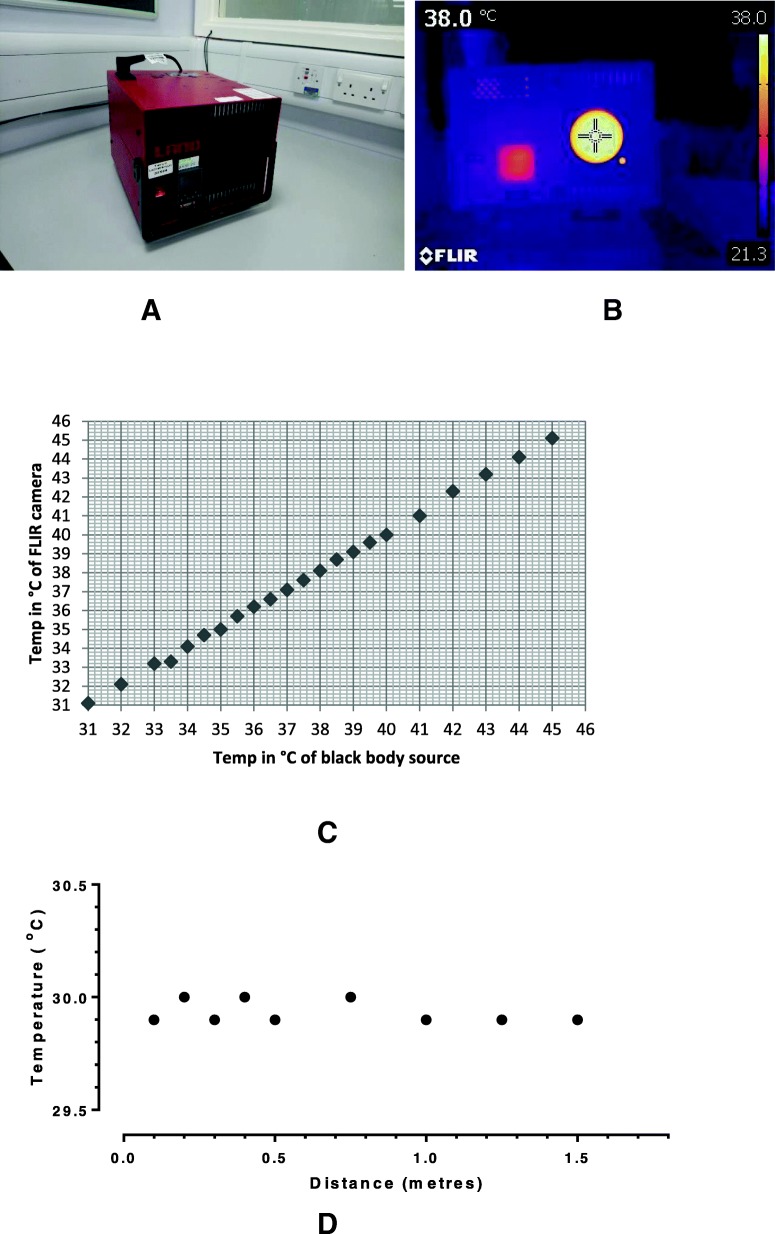
Bench-top black body instrument (a) set to 38 °C showing equivalent temperature measured with the camera lens positioned in front of the heat source and with pixel value obtained (at cross-hair) of 38 °C (b). Graph (c) shows results for reliability of the thermal camera across a temperature range (31-46 °C) revealing consistency between camera and black body source to within + 0.1^o^ to 0.2 °C. Distance of camera lens from black body source has minimal effect on temperature readings (d)

The thermal imaging camera was calibrated between 30 °C and 45 °C; against a black body source (Fig. 2 a, Fig. 2b; P80P, Ametek-Land, Dronfield, UK) During calibration, temperature measurements from the thermal camera (T450sc, uncooled microbolometer, FLIR, Täby, Sweden) were compared (Fig. 2c) to measurements from a certified (UKAS, UK) independent thermometer, of type 100 Ω platinum resistance thermometer (PRT100, ISOTECH, Skelmersdale, UK) in situ within the black body system.

### Measurement uncertainty

The measurement uncertainty of the FLIR T450sc camera was evaluated as the root mean squared error (RMSE) metric of the measurements acquired during calibration. This allowed uncertainties to be assigned to the patient temperature measurements during the course of the study.

### Effect of distance

To determine if the distance of the camera and focus has any effect on the constant temperature detected from the target a separate series of experiments were undertaken. Here, the black body system was heated, as before, to 30 °C and allowed to stabilise for 10 min. Temperature measurements were taken using the FLIR camera at distances from the black body source ranging from 0.1 m to 1.5 m (Fig. 2d).

### Visual assessment of wounds

From the digital photographs obtained from the participants at each of the four imaging sessions, six senior clinicians (midwife, doctor, nurse) affiliated to the study (but ‘blind’ to patient identifiers, thermal image analysis and the wound outcome) rated each woman’s risk of wound infection at days 2 and 7. The arithmetic mean of Cohen’s kappa for all pairs of observers was used as a measure of inter-observer agreement on each day. At both time-points ‘observers’ were asked to rate the appearance of the c-section scar as ‘yes’; ‘no’; or ‘uncertain’ as to the chance of developing an SSI within 30 days (agreement on prediction of SSI).

### Data analyses

The sample was summarised descriptively (SPSS *ver* 24). A series of univariate logistic regression models were derived to model the dichotomous outcome of SSI occurrence by day 30. Each model considered one of the following candidate (early) predictor variables: abdominal temperature at day 2 postpartum (*ROI 1 D2*); abdominal temperature at day 7 postpartum (*ROI 1 D7*); wound temperature at day 2 postpartum (*ROI 2 D2*); wound temperature at day 7 postpartum (*ROI 2 D7*); wound-abdominal temperature difference at day 2 postpartum (*WATD-2*); wound-abdominal temperature difference at day 7 postpartum (*WATD-7*). Information of the regions of interest (as for days 2 and 7) were also collected at Day 15 and Day 30 but were not used in subsequent regression analyses, as assessment of the early prediction of SSI was the objective.

The extent of any correlation between the same measure determined at different time points, and between different measures determined at the same time points, was assessed.

Cohen’s kappa statistic was used to measure levels of agreement between all six clinician raters’ assessments made on day 2 and again for day 7. Pairwise kappa statistics were also used to assess agreement on opinion of wound assessment for signs of SSI from photographs taken on day 2 and again on day 7.

ROC curves were constructed for all models with significant predictors. The area under the ROC curve (AUROC) statistic, with 95% confidence intervals, was derived for each curve, with an optimum cut-off at stated values of sensitivity and specificity.

## Results

### Calibrations

The FLIR thermal camera calibration (Fig. 2c) demonstrated that the device measured consistently + 0.1 to + 0.2 °C higher than the black body source thermometer (set to 30 °C) when used in an open environment. At each of the selected distances (0.1 to 1.5 m between camera lens and black body), the source temperature did not vary by more than 0.1 °C (Fig. 2d).

### Measurement uncertainty

The RMSE of the black body source temperature measurement was evaluated to be ±0.53 °C from comparison with the temperature, measured using a transfer standard platinum resistance thermometer (PRT) inserted into the calibration thermo-well of the black body source (Fig. 2b). The RMSE of the camera calibration was estimated to be ±0.13 °C by comparison with the black body-measured temperatures. The overall uncertainty around the camera measurements was estimated to be ±0.55 °C. The uncertainty of the PRT was certified by the manufacturer to be an order of magnitude smaller than the RMSE values calculated for the measurements, and therefore was neglected in evaluating the overall measurement uncertainty.

### Participants

Fifty three women were recruited to the study (Table 1). Of these, 3 women were discharged before imaging could be undertaken; 50 women, aged 21 to 44 years (median 32 years), with a BMI within the range of 30.1 kg⋅m^− 2^ to 43.9 kg⋅m^− 2^ (median 34.2 kg⋅m^− 2^) entered the study. At the time of first thermal imaging, 1–3 (median 2) days after surgery, all women were afebrile with aural temperatures ranging from 36.2 °C to 37.3 °C (median 36.8 °C). Of the 50 women, 14 (28%) were confirmed with a diagnosis of SSI. The sample is summarised fully in Table 1; partitioned by SSI status by day 30, and as an entire cohort.

**Table 1:** Patient characteristics

Categorical factor	Frequency (valid %)		
	SSI (*n* = 14)	Non-SSI (*n* = 36)	All patients (*n* = 50)
Procedure			
Planned	6 (42.9%)	14 (38.9%)	20 (40.0%)
Emergency	8 (57.1%)	22 (61.1%)	30 (60.0%)
Ethnicity			
White British	13 (92.9%)	25 (69.4%)	38 (76.0%)
Non-White British	1 (7.1%)	11 (30.6%)	12 (24.0%)
Number of pregnancies			
1	2 (14.3%)	14 (38.9%)	16 (32.0%)
2	4 (28.6%)	7 (19.4%)	11 (22.0%)
3	5 (35.7%)	7 (19.4%)	12 (24.0%)
4	1 (7.1%)	5 (13.9%)	6 (12.0%)
5	0 (0.0%)	2 (5.6%)	2 (4.0%)
6	1 (7.1%)	1 (2.8%)	2 (4.0%)
7 or more	1 (7.1%)	0 (0.0%)	1 (2.0%)
Number of live births			
1	2 (14.3%)	18 (50.0%)	20 (40.0%)
2	10 (71.4%)	6 (16.7%)	16 (16.7%)
3	1 (7.1%)	7 (19.4%)	8 (16.0%)
4	0 (0.0%)	4 (11.1%)	4 (8.0%)
5	0 (0.0%)	1 (2.8%)	1 (2.0%)
6	0 (0.0%)	0 (0.0%)	0 (.0.0%)
7 or more	1 (7.1%)	0 (0.0%)	1 (2.0%)
Number of C-sections (*n* = 49)			
1	5 (38.5%)	25 (69.4%)	30 (61.2%)
2	6 (46.2%)	7 (19.4%)	13 (26.5%)
3	2 (15.4%)	2 (5.6%)	4 (8.2%)
4	0 (0.0%)	2 (5.6%)	2 (4.1%)
Variable	Mean (SD)		
	SSI (*n* = 14)	Non-SSI (*n* = 36)	All patients (*n* = 50)
Number of C-sections (*n* = 49)	1.77 (0.725)	1.47 (0.845)	1.55 (0.818)
Blood loss (ml) (*n* = 50)	472 (149)	692 (364)	630 (332)
Pre-operative haemoglobin (g/L) (*n* = 49)	114 (8.03)	119 (13.7)	117 (12.5)
Post-operative haemoglobin (g/L) (*n* = 43)	101.4 (8.73)	101 (11.8)	101 (11.0)
Pre-operative white blood cell count (×10^9^/L) (*n* = 48)	10.7 (3.91)	11.1 (3.87)	11.0 (3.84)
Post-operative white blood cell count (× 10^9^/L (*n* = 46)	10.9 (5.09)	13.1 (3.81)	12.5 (4.23)
Gestational age (weeks)	39.7 (1.28)	39.2 (1.67)	39.4 (1.53)
Body mass index (kg.m^−2^)	35.2 (3.89)	35.3 (3.99)	35.3 (3.92)
Body temperature (°C) (*n* = 50)	36.8 (0.31)	36.8 (0.25)	36.8 (0.26)
Abdominal temperature Day 2 (°C) (*n* = 50)	33.4 (0.79)	33.8 (0.80)	33.7 (0.81)
Wound temperature Day 2 (°C) (*n* = 50)	35.1 (0.53)	34.9 (0.69)	35.0 (0.65)
Abdominal temperature Day 7 (°C) (*n* = 47)	32.5 (0.98)	33.5 (0.98)	33.2 (1.07)
Wound temperature Day 7 (°C) (*n* = 47)	34.4 (0.77)	34.5 (0.80)	34.5 (0.78)
Abdominal temperature Day 15 (°C) (*n* = 44)	31.7 (1.87)	32.6 (1.01)	32.3 (1.36)
Wound temperature Day 15 (°C) (*n* = 44)	33.7 (1.11)	34.1 (0.86)	34.0 (0.95)
Wound minus abdominal temperature difference Day 2 (°C)	1.73 (0.96)	1.12 (0.77)	1.29 (0.86)
Wound minus abdominal temperature difference Day 7 (°C) (*n* = 47)	1.92 (1.00)	1.09 (1.01)	1.34 (1.07)
Wound minus abdominal temperature difference Day 15 (°C) (*n* = 44)	1.96 (1.59)	1.56 (1.29)	1.68 (1.36)

Exploring for the raters’ opinions in assessing the wound on day 2 for likely SSI, Kappa statistics between all six raters ranged from 0.155 to 0.556 (mean 0.329); hence the levels of agreement ranged from slight to moderate. At day 7, pairwise Kappa statistics between all six raters ranged from 0.223 and 0.670 (mean 0.428) suggesting a slight improvement in agreement at day 7 over day 2, but still moderate agreement only.

From the wound photographs on day 2, a visual assessment was made by the 6 clinician raters as to the likelihood of an SSI. Results showed poor agreement with the subsequently confirmed outcome. Pairwise Kappa statistics ranged from − 0.124 to 0.086 with a mean of − 0.026, indicating no improvement over chance agreement for the raters’ wound assessment for likely SSI. By day 7, pairwise Kappa statistics between individual raters’ and eventual wound infection diagnoses ranged from − 0.040 to 0.190 with a mean of 0.112, indicating minimal improvement over the Day 2 levels of agreement between the clinician raters.

Among the six raters reviewing the wound photographs on day 2, prediction rates for later diagnosis of infection were between 2 and 30%. Similarly, on day 7, the six raters diagnosed likely infection in between 4.1 and 26.5% of cases. This suggests that prevalence of SSI in the sample will impact upon the estimate of Kappa and may cause wound infection to be under- or over-estimated.

### Thermal imaging in hospital and at community follow-up

In this imaging series, thermal images were taken on 4 occasions, one in hospital before discharge and 3 at home. In 39 (78%) women, a full set of images (taken on four different days) were obtained. In 11 cases, access was not available on some of the follow-up days.

### Ambient temperature

Ambient conditions (air temperature, relative humidity (RH) %) of the postnatal unit ranged from 20.9 °C to 27.4 °C (median 24.0 °C) air temperature and 39 to 73% (median 53%) RH. At the second, third and fourth follow-up visits (nominally, days 7,15, 30), air temperature ranged from 17.4 °C to 25.8 °C (median 21.6 °C), from 17.3 °C to 26.8 °C (median 21.3 °C), and from 15.7 °C to 25.6 °C (median 20.9 °C) respectively; RH ranged from 39 to 73% (median 53)%, from 41 to 77% (median 58%) and from 42 to 72% (median 58%) respectively. There was no effect of ambient temperature or RH on skin temperature measurements across the measurement intervals.

### Post-operative oral antibiotic prophylaxis

With one exception (where *i.v* benzylpenicillin 1.8G was administered), all patients received the standard intra-operative, *i.v* antibiotic regimen (Cefuroxime 1.5G, Metronidazole 500 mg *stat*). In addition, post-operative oral antibiotic prophylaxis was given to 19 women (38%), 17 of whom (34%) were considered at SSI risk). Two women received post -operative antibiotics for urinary tract infections. Eight of 17 women (47%) received antibiotic prophylaxis due to high SSI risk (obese category 3; BMI ≥40 kg⋅m^− 2^). Nine of 17 women (53%) in obese categories 1 and 2 (BMI 35 kg⋅m^− 2^ to 39.9 kg⋅m^− 2^) also received prophylactic antibiotics.

### Wound assessment and antibiotic administration during community follow-up

At the 30-day post-operative time-point, wound outcome was followed up with the GP. Sixteen women (32%) returned to their GP, between 6 and 24 days after caesarean section (median 18 days); one woman returned on two occasions. Of the 16 women returning to their GP, the reason was for a suspected wound infection or delayed healing; 14 women (28%) were prescribed oral antibiotics, with a corresponding clinical diagnosis of SSI. Two woman received antibiotics but no SSI diagnosis.

Wound swabs were not taken during in-patient stay but in 14 women (28%), a wound swab was taken at the time of the GP visit. Gram positive organisms (*Streptococcus species*, group B) were isolated in one woman only. The remainder of isolates were either moderate or heavy growth of anaerobes. Four of 50 women (8%) with a GP confirmed SSI received i.v intraoperative antibiotics, immediate oral antibiotics and, subsequently, one or more courses of oral antibiotics.

### Abdominal thermography and ROI mapping

Values for mean abdominal temperature and for wound site fell over time, from Day 2 to Day 30, but with wound site temperature remaining approximately 1.5 °C higher than abdominal temperature at each imaging session (Table 1). Differences in the temperature maps of both regions were observed between those patients who developed a wound infection by comparison to those who did not (Table 1).

#### Modelling of abdominal temperature

Mean abdominal temperature (ROI 1) was lower on days 2, 7 and 15 postpartum in women who subsequently developed a wound infection by Day 30 compared with those who did not (Table 1). Logistic regression models conducted on the ROI 1 measure at Days 2, 7 and 15 postpartum (Table 2) revealed that lower abdominal temperature was significantly associated, at the 5% significance level, with an increased risk of infection (*p* = 0.011) at day 7; with substantive but non-significant associations also observed at Day 2 and Day 15.

**Table 2:** Univariable logistic regression parameters

Variable	Day	*p*-value	Odds ratio (OR)	95% CI for OR	Cases correctly classified	Nagelkerke’s pseudo-R^2^
ROI 1	2	0.112	0.51	(0.222, 1.17)	76.6%	0.076
	7	0.011	0.365	(0.168, 0.793)	70.5%	0.237
	15	0.070	0.613	(0.364, 1.04)	72.7%	0.117
ROI 2	2	0.331	1.62	(0.610, 4.31)	72.0%	0.028
	7	0.609	0.813	(0.362, 1.82)	70.2%	0.008
	15	0.135	0.588	(0.292, 1.18)	72.7%	0.073
(ROI 2-ROI 1)	2	0.034	2.25	(1.07, 5.15)	70.0%	0.140
	7	0.023	2.45	(1.13, 5.29)	78.7%	0.186
	15	0.388	1.23	(0.769, 1.98)	72.7%	0.024

At day 7, a 1 °C increase in mean abdominal temperature was associated with an odds ratio (OR) of infection of 0.365 (95% CI: 0.168 to 0.793); i.e. a unit (i.e.1 °C) decrease in abdominal temperature was associated with approximately a 3-fold raised odds of SSI at best estimate. Despite the higher level of significance associated with the ROI 1 measure at Day 7, the number of cases correctly classified by this model was lower than at Day 2 or Day 15; however, the proportion of cases classified correctly was very similar at all three measured time points (70.5 to 76.6%).

At day 7, the mean difference in abdominal temperature between those with an SSI diagnosis and those with no SSI diagnosis was 0.95 °C (95% CI: 0.32, 1.58 °C) lower in those who subsequently developed a wound infection. This difference between the two groups was significant at the 5% significance level (*p* = 0.004).

The ROI 1 model at Day 7 was a good fit to the data (Nagelkerke’s pseudo-R^2^ = 0.237); other models were revealed to have moderate goodness-of-fit to the data.

#### Modelling of wound site temperature

Mean wound site temperature (ROI 2) was similar at Days 2, 7 and 15 postpartum in patients who subsequently developed an SSI to those who did not (Table 1). Logistic regression models conducted on the ROI 2 measure at Days 2, 7 and 15 postpartum (Table 2) revealed that lower wound temperature was associated with a substantive, but non-significant increased risk of infection (*p* = 0.135) at day 15; and not substantively or statistically associated with infection at Day 2 or Day 7. The proportion of cases classified correctly was very similar at all three measured time points (70.2 to 72.7%); and also similar to those obtained from the use of the ROI 1 measure.

The ROI 2 model at Day 15 was a moderate fit to the data (Nagelkerke’s pseudo-R^2^ = 0.073); other models were revealed to have poor goodness-of-fit to the data.

#### Modelling of the wound site temperature – Abdominal temperature difference

Mean wound site temperature-abdominal temperature difference (WATD) on days 2, 7 and 15 postpartum was greater in patients who develop a wound infection (Table 1).

Logistic regression models conducted on the WATD measure at Days 2, 7 and 15 postpartum (Table 2) revealed that greater temperature difference was significantly associated at the 5% significance level with an increased risk of infection at Day 2 (*p* = 0.034) and at Day 7 (*p* = 0.023); and not substantively or statistically associated with infection at Day 15. At day 2, a 1 °C wider difference between mean wound site temperature and abdominal temperature was associated with an odds ratio (OR) of infection of 2.25 (95% CI: 1.07 to 5.15); i.e. a unit (i.e. 1 °C) widening of temperature between wound and abdomen was associated with approximately a 2-fold raised odds of SSI at best estimate. At day 7, a 1 °C increase in the mean wound site - abdominal temperature difference was associated with an odds ratio (OR) of infection of 2.45 (95% CI: 1.13 to 5.29); i.e. a unit (i.e. 1 °C) increase in temperature difference was associated with approximately a 2.5-fold raised odds of SSI at best estimate.

The proportion of cases correctly classified by these models were similar to the proportion correctly classified by the models based on single-location temperatures with the optimum classification (78.7% correct) achieved at Day 7.

Despite the higher level of significance associated with the ROI 1 measure at Day 7, the number of cases correctly classified by this model was lower than at Day 2 or Day 15; however, the proportion of cases classified correctly was very similar at all three measured time points (70.5 to 76.6%).

The WATD model at Days 2 and 7 was a moderately good fit to the data (Nagelkerke’s pseudo-R^2^ = 0.140 at Day 2; and 0.186 at Day 7); the model based on data collected at Day 15 fitted less well to the data.

Correlational analysis revealed the existence of moderate positive correlation between the ROI 1 measures extracted at Day 7 and Day 15. The ROI 2 measures taken at each time point were all positively moderately correlated with each other. Moderate positive correlations were also observed between the ROI 1 and ROI 2 measures at each time point.

### ROC analyses

**Fig. 3:**
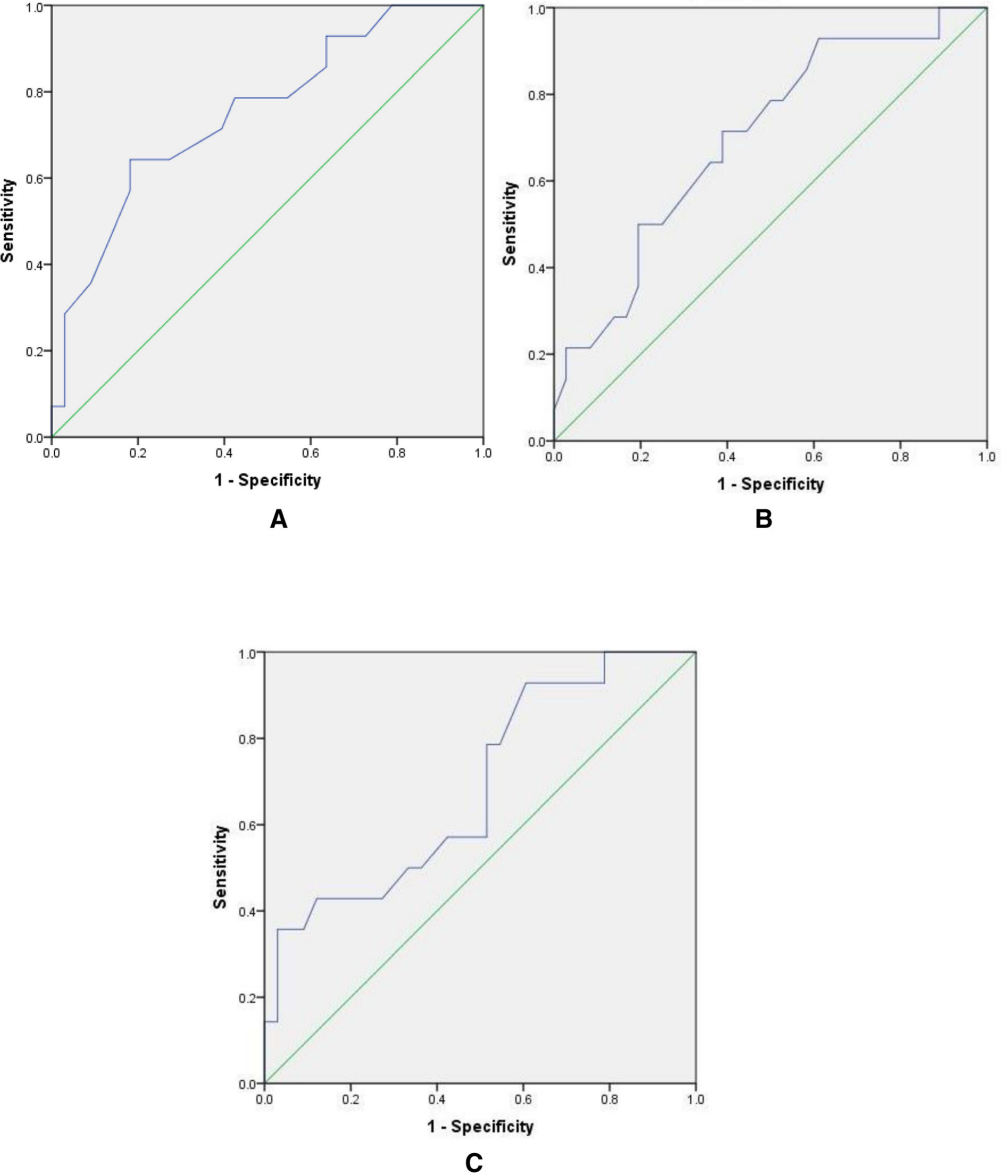
Predictive capability of statistical models with receiver operator curve plots of sensitivity versus 1-specificity for abdominal temperature at day 7 (a), wound minus abdominal temperature difference at day 2 (b), and wound minus abdominal temperature difference at day 7 (c)

An ROC analysis conducted for the ROI 1 measure extracted at Day 7 revealed that the AUROC statistic for this parameter was 0.752 (95% CI: 0.599 to 0.905), representing good predictive capability. A suitable cut-off focussing on optimising sensitivity was given by 33.9 °C, corresponding to 92.9% sensitivity and 36.4% specificity. A suitable cut-off focussing on optimising specificity was given by 32.65 °C, corresponding to 64.3% sensitivity and 81.8% specificity (Fig. 3a).

An ROC analysis conducted for the difference measure extracted at Day 2 revealed that the AUROC statistic for this parameter was 0.697 (95% CI: 0.538 to 0.857), representing fairly good predictive capability. A suitable cut-off focussing on optimising sensitivity was given by 0.75 °C, corresponding to 92.9% sensitivity and 38.9% specificity. A suitable cut-off focussing on optimising specificity was given by 1.7 °C corresponding to 50.0% sensitivity and 80.6% specificity (Fig. 3b).

An ROC analysis conducted for the difference measure extracted at Day 7 revealed that the AUROC statistic for this parameter was 0.687 (95% CI: 0.521 to 0.854), representing fairly good predictive capability. A suitable cut-off focussing on optimising sensitivity was given by 0.85 °C, corresponding to 92.9% sensitivity and 39.4% specificity. A suitable cut-off focussing on optimising specificity was given by 2.40 °C, corresponding to 35.7% sensitivity and 95.9% specificity (Fig. 3c).

## Discussion

Protecting and improving the health of a nation is the core business of all health systems. In the United Kingdom, the National Health Service (NHS) ‘serves’ a million patients every 36 h with forecasted costs for 2018/19 of over £126bn [29]. With 1 in 10 of the population undergoing surgical procedures, over 5 million of which are undertaken under anaesthesia in an operating theatre, the number of surgeries are increasing year on year; with a 4.2% increase from 2009 to 2014 [29].

Although post-operative mortality is decreasing [30], there is considerable variability in survivor outcomes. To an extent, understanding the impact of surgery and the outcomes or quality of care delivered to a Nation’s health service is now being addressed (in the UK) via patient reported outcomes (PROMS) programme. Surgical site infection surveillance is currently limited to just four surgical categories [31]. At present, SSI reporting after c-section is neither a mandatory nor a voluntary requirement of the surgical site infection surveillance service (SSISS) [31]. It may not be surprising therefore that the incidence of SSI after c-section has such variation in reported incidence. The lack of a gold standard for SSI assessment persists as a ‘gap’ in our ability to measure and monitor wound infections. Bruce et al. (2001) reports 41 different definitions of SSI and 13 grading scales to describe wound infection in a systematic review of 82 included studies [32]. A definitive assessment of the surgical wound is overdue.

In this study we have taken the first steps towards investigating whether independent, quantitative wound imaging, as a non-invasive, non-ionising technology, has the potential to stratify patients (on the basis of the acquired thermal signature) to the later development of SSI. With acceptable performance, this technology has potential wide-reach and global impact for SSI risk stratification. Importantly, as we enter what is currently described as a ‘post-antibiotic era’ [33], concerns about a crisis in antibiotic resistance [34], attributed to the overuse of antibiotics, makes the need for rational antibiotic prescribing, now a part of the wider conversation in the media and community, ever more important.

In this study 14 of 50 obese women, presenting to the clinic on average18 days after c-section, were prescribed antibiotics for surgical wound infection, This SSI rate of 28% is higher than that reported for high risk ‘dirty’ colorectal wounds [35] exceeds the 18% reported by Jasmin et al [18] in a mixed BMI population but lower than reported by our previous study in morbidly obese women [20]. Whether this SSI was truly due to pathogens residing in superficial tissue causing delay in wound closure or even to superficial wound dehiscence (SWD) in the absence of infection remains unclear [36]. Intra-operative intravenous antibiotic prescribing was 100% with 38% additional (oral) courses prescribed post-operatively (within the first two days before hospital discharge). Even so 28% of women were reported to have developed an SSI. How often is clinical suspicion of SSI accurate? How frequently are antibiotics being prescribed, just in case? Without a ‘gold’ standard for SSI diagnosis it is not possible to tell. Even if wound swabs are taken, the methods used are notoriously unreliable in capturing bacteria [37]. Furthermore, swab results per se are not diagnostic of infection; bacterial load virulence and host factors all play a role in eventual susceptibility to infection [38].

Diagnosis of SSI in this study was based on visual assessment of the wound by the GP following a visit to clinic. Where wound swabs were taken (and with the exception of one wound swab positive for Group B *Streptococcus*) laboratory confirmation was of either ‘no growth’ or ‘moderate to heavy growth’ of anaerobes. As anaerobic bacteria make up a significant proportion of the normal microbiota colonizing skin and various mucosal surfaces of the human body [39] it is not clear which, if any, pathogenic species were present in the sample. Furthermore, antibiotics are typically prescribed *before* laboratory results are known. Two key factors emerge; the rate of SSI remains a clinical diagnosis, and antibiotics continue to be prescribed to treat infection even without knowledge of a causative organism. In seeking to obtain an objective technique to assess the wound and to stratify women to SSI risk on the basis of biological, rather than anthropometric, risk we have taken steps ‘towards’ a gold standard for antibiotic prescribing by developing an objective method to determine if there is an early (thermal) signature in those at highest risk of SSI. Here we used infrared thermography to image the wound and adjacent abdominal structures.

Infrared energy is dependent upon radiation energy emitted from the skin (which is proportional to heat generated) [40] but invisible by eye. With modern thermal cameras it is possible to ‘see’ infrared energy as a temperature map together with absolute values for temperature given appropriate corrections for skin emissivity [41]. Recent studies have confirmed the relationship between human skin temperature, measured using thermography, and perfusion (measured using laser speckle contrast imaging). The different techniques show high convergent validity making thermography a robust surrogate for skin blood flow [42, 43]. As observed in the present study, a consistent increase in wound site temperature (ROI2) of approximately 1.5 °C was evident over the course of 15 days (and including day 30 measures) compared to non-injured abdominal skin. This increase in wound temperature fits well with the long-held observation of a local increase in blood flow consequent upon local tissue inflammation. This temperature increase and associated blood flow has been used as a diagnostic sign of wound infection [40]. In the present series, we have observed a temporal wound profile which remains elevated above the temperature of the abdomen consistently over the first month in those with, as well as in those without SSI, so there seems little prognostic value in thermography of the wound site per se for predicting those patients who later go on to develop SSI. However, wound site measures, in conjunction with the temperature of the abdomen, affords promise as a predictor of subsequent SSI. Of note here is the significantly wider temperature gradient between wound and abdominal temperatures on days 2 and 7, a time-point which precedes SSI onset. With ambient temperature having no significant effect on skin temperature values at the four measurement intervals and with mean wound temperature similar at early time points (days 2,7,15) the widening of the temperature gradient is primarily due to low abdominal temperature. In this study, we report a new observation that the temperature of the tissue adjacent to the wound site may play a more important role in risk for SSI and one possible explanation as to the link between obesity as a biological risk factor for SSI. As early as day 2 and day 7 we observed a 2.25 and 2.45 odds, respectively, for SSI with each 1 °C widening of the temperature gradient between wound site and abdomen.

We have observed previously, albeit in a South East Asian population [25], that mean abdominal temperature is inversely related to BMI category; lowest temperature values in participants with the higher BMI. Since subcutaneous fat has low thermal conductivity, an increase in abdominal fat effectively insulates the body [44] and interferes with heat transfer from body core, so lowering skin temperature [45]. Obesity, a condition associated with a high body heat content [46] and higher resting energy expenditure, compared to lean individuals is primarily due to a larger fat-free (muscle) mass [45]. However, due to increased body insulation and reduced conductive heat flow via the trunk, acral regions [47] (hands and feet) become major sites for heat dissipation in the face of an increased metabolic heat production. Savastano et al. [45] using infrared thermography show the effect of abdominal adiposity between normal weight and obese women; thermal mapping revealing a significant (1 °C) reduction in mean abdominal temperature in the obese compared to the normal-weight group. There is the possibility that reduced blood flow in the region surrounding the wound incision may compromise the delivery of oxygen and nutrients to the wound, so adding a risk for slow wound healing or infection by creating areas of skin commensurate with ‘low perfusion’. Another possibility is of a ‘dead-space’ vascular region possibly due to seroma or oedema. We have shown previously that after colorectal surgery [25] and c-section [26] temperature ‘cold spots’ [48] along the wound on thermography are observed in patients with confirmed SSI. As illustrated in Fig. 1B, ‘cold spots’ (low radiation intensity) were also evident in some women, at times, in the present study. However, located within a wound region of higher average temperature (and blood flow), by averaging the temperature values, the cold spots are masked on quantitative analyses. The qualitative thermal map as well as arithmetic mean of the ROI’s must be considered as complimentary for SSI risk prognosis. Further investigations to co-locate the extent and number of ‘islands’ of low temperature within the wound, together with the contribution of the adjacent (healthy) abdominal temperature per se will improve the sensitivity of the predictive model. Currently, whilst not outstanding in any of the models, the low number of cases in the sample (but a size appropriate for an early-stage pilot study) precludes the derivation of multiple models in the current analysis. None of the observed correlations suggested that a multiple model would be subject to excessive co-linearity; hence future modelling could investigate the potential of the inclusion of multiple temperature measurements in a logistic regression model to improve predictive capability. The predictive capability of abdominal temperature (ROI 1) and temperature differences (wound site – abdominal temperature; WATD) is substantially better than the assessment by the clinician’s; which was in general no better than chance.

One of the limitations in undertaking this study is that we were unable to establish, with accuracy, the depth of SSI but, based upon the clinical assessment as well as review of photographs, it is likely that, in this series at least, the majority of wounds were superficial SSI’s as none required wound debridement, referral to a plastic surgeon or prolonged hospital admission requiring prolonged intravenous antibiotics.

On the matter of the choice of follow up days to undertake post-operative thermal imaging as a predictive technique for SSI, we had little prior knowledge of the peak onset of SSI in this population. Our objective therefore was to assess and image the wound as often as practical within the first 30 days of surgery. A pragmatic decision was taken to optimise the number of community visits to three and to determine the day of onset of SSI from the GP per se. We have shown that the earliest time-point with best predictive performance is within 7 days after surgery, the timepoint at which 98% of women had not yet complained of a wound problem; all but one of the women developed SSI after day 7. As the median time of SSI diagnosis was at day 18, imaging up to day 7 ‘captures’ the predictive interval before clinical signs appear.

The question remains, should we *and* can we, use antibiotics more sparingly in this era of AMR? As recommended by Lord Jim O’Neill, Chair of the Review on Antimicrobial Resistance [49] a step change is needed in diagnostic technology. This includes not only the development of faster laboratory methods and ongoing search for new classes of antibiotics, but also a more reliable method for wound assessment independent of subjective opinion, which in this study was shown to be highly variable between clinicians. In attempting to achieve an objective (infrared) thermography technique for ‘looking at wounds’ we have developed promising performance of a technology towards SSI risk prognosis and defining of a biological signature for surgical wound infection.

## Conclusions

In this study cohort of women with a BMI ≥35.0 Kg.m^− 2^, commensurate with a weight category of obesity, 28% of women were clinically diagnosed with a SSI 2–3 weeks after surgery. Wound infections developed in over one quarter of the cohort despite prodigious use of intravenous and oral antibiotic prophylaxis.

Visual assessment of the wound was poor for there was a lack of agreement between clinicians in their subjective assessment of the wound and a lack of agreement in identifying the wounds most likely to become infected. However, by using quantitative imaging and thermal mapping of the wound and abdomen at early times (day 2 and day 7) after surgery, improved predictions for later SSI was achieved in 77 and 70% of cases respectively. Best fit of the model was at day 7 from the temperature difference between wound and abdomen. The wider the temperature difference between the sites, the greater the odds of infection. With wound temperature remaining relatively constant across the first 15 days after surgery, it is the contribution of a low abdominal temperature that makes a substantive (at day 2) and significant three-fold (day 7) contribution to an increased risk of infection in obese women during the first week after surgery.

With a larger cohort of participants, verification of the predictive performance of infrared thermography signatures reported here offers potential for the development of a non-invasive, low-cost imaging modality. Augmenting subjective assessment with thermographic wound imaging would provide a rational approach to determine those women most at risk of SSI and thus those most in need of antibiotics. This would be of significant benefit in this obese population. Currently antibiotics are administered prophylactically by clinical protocol based on BMI range rather than SSI risk.

### Implications for research and practice

Considering the associated risks of morbidity from undetected infections and the added risks of antibiotic resistance in leading to longer hospital stays, higher medical costs and increased mortality, this technology could reduce the cost and resource burden on health service provisions. Within the obstetric field this could also have a significant impact on improving postpartum recovery experience for mothers and their families, given the high and increasing rates of both obesity and caesarean sections globally.

With a larger cohort of participants, verification of the predictive performance of infrared thermography signatures reported here offers potential for the development of a non-invasive, low-cost imaging modality. Augmenting the existing visual subjective assessment with thermographic wound imaging would provide a more reliable approach to determine those women most at risk of SSI and thus those most in need of antibiotics.

## Abbreviations

AMR: Antimicrobial resistance
AUROC: Area under the ROC curve
BMI: Body mass index (Kg.m^− 2^)
CDC: Centre for Disease Control
CRN: Clinical Research Network
D2, D7: Day 2, day 7
FOV: Field of view
GP: General practitioner
HAI: Hospital acquired infection
IR: Infrared
IRT: Infrared thermography
NHS: National Health Service
NIHR: National Institute for Health Research
NPWT: Negative pressure wound therapy
OR: Odds ratio
PROMS: Patient reported outcome measures
PRT: Platinum resistance thermometer
RMSE: Root mean squared error
ROC: Receiver operator characteristic
ROI: Region of interest
SE Asian: South East Asian
SPSS: Statistical package for the social sciences
SSI: Surgical site infection
SSISS: Surgical site infection surveillance service
UK: United Kingdom
WATD: Wound-abdominal temperature difference
